# PET Imaging of Diabetes-Induced Alterations in Metabolism and Immune Activation

**DOI:** 10.1007/s11307-025-02027-y

**Published:** 2025-08-12

**Authors:** Shannon E. Lynch, Heba M. Alsheikh, Patrick N. Song, Candace C. Parker, Yujun Zhang, Clayton C. Yates, Benjamin M. Larimer, Suzanne E. Lapi, Lalita A. Shevde, Anna G. Sorace

**Affiliations:** 1 Department of Radiology, The University of Alabama at Birmingham, VH G082, 1670 University Blvd, Birmingham, AL 35233 USA; 2 Department of Biomedical Sciences, The University of Alabama, Birmingham, Birmingham, AL USA; 3 Department of Pathology, The University of Alabama at Birmingham, WTI 320D, 1824 6th Ave S, Birmingham, AL 35233 USA; 4 Department of Radiology, Memorial Sloan Kettering Cancer Center, New York, NY USA; 5 Department of Pathology, Johns Hopkins University, Baltimore, MD USA; 6 O’Neal Comprehensive Cancer Center, The University of Alabama at Birmingham, Birmingham, AL USA; 7 Department of Chemistry, The University of Alabama at Birmingham, Birmingham, AL USA; 8 Department of Biomedical Engineering, The University of Alabama at Birmingham, Birmingham, AL USA

**Keywords:** Type 2 diabetes, Obesity, CD206, FDG, Granzyme B, Breast cancer

## Abstract

**Introduction:**

Obesity and type 2 diabetes (T2D) influence the tumor microenvironment by altering glucose metabolism, which has been shown to decrease immune cell infiltration and activation. Positron emission tomography (PET) imaging provides a non-invasive method to detect molecular markers of immune populations in the tumor microenvironment and systemic organs. The goal of this study is to utilize advanced molecular imaging to quantify differences in innate and adaptive immune responses in diabetic obese mice systemically and within the tumor microenvironment.

**Methods:**

5–6-week-old female C57BL6/J mice were placed on a high-fat diet (HFD) composed of 60% kcal fat or control low-fat diet with 10% kcal fat. Animals were treated with subsequent low doses of streptozotocin to induce T2D and blood glucose was monitored. Following induction of diabetes, E0771-luc + cells were implanted into the 4th mammary fat pad and allowed to grow to a tumor volume of 100mm^3^. PET imaging was acquired over the course of 5 days with the following tracers: [^18^F]-FDG PET for glucose metabolism, [^68^Ga]Ga-RP832c (CD206) PET for M2 macrophages, and [^68^Ga]Ga-GZP PET for granzyme B, an indicator of effector cell activation, and [^18^F]-DPA-714 PET for neuroinflammation. Regions of interest were identified for the tumor, brain, kidneys, heart, muscle, brown adipose tissue (BAT), to characterize differences in important organs and tumor tissue. Metrics of standardized uptake value (SUV) were extracted from imaging data including mean, max, peak, and tumor-to-background ratios. Following the final imaging timepoint, tumors were extracted for biological characterization via flow cytometry.

**Results:**

Diabetic obese mice have no difference in tumor glucose metabolism, but have decreased FDG uptake in the brain and BAT compared to controls. Obesity and T2D systemically affect innate and adaptive immune infiltration and activation including significantly increased RP832c and GZP in muscle, heart, brain, and BAT. Hyperglycemic tumors had trending decreases in GZP SUV_mean_ and increased RP832c SUV_mean_. Flow cytometry shows diabetic obese tumors have a significant increase in CD206 + macrophages and no significant difference in GZB + CD8 + T cells compared to controls.

**Conclusion:**

PET imaging reveals that obesity and T2D alter glucose metabolism and immune activation while suppressing tumor-immune activation in diabetic obese mice both within the tumor microenvironment and systemically.

**Supplementary Information:**

The online version contains supplementary material available at 10.1007/s11307-025-02027-y.

## Introduction

Over the last decade, there has been a rise in obesity and type 2 diabetes (T2D) globally, with the World Health Organization declaring each as an epidemic due to their high prevalence [1]. Incidence of these conditions is rising in the U.S., where more than two-thirds of adults are estimated to be overweight or obese, as defined by their body mass index (BMI) [2]. Obesity is a chronic inflammatory condition associated with metabolic diseases including diabetes, cancer, cardiovascular disease, and other comorbidities [2–4]. There is a strong relationship between diabetes and weight, with over 90% of patients with T2D considered to be overweight (BMI ≥ 25 kg/m^2^) or obese (BMI ≥ 30 kg/m^2^) [5]. Obesity and diabetes are associated with hyperinsulinemia, hyperglycemia, dyslipidemia, adiposity, and inflammation, which increase patients’ risk for developing numerous cancers including breast cancer [1, 6, 7]. Patients with obesity and diabetes often develop more aggressive subtypes of breast cancer, including triple-negative breast cancer (TNBC), and have worse disease recurrence, and increased mortality [8–10].

It is well-established that cancer cells have increased uptake of glucose compared to normal cells and primarily utilize aerobic glycolysis to produce energy [11]. Hyperglycemia, a diagnostic hallmark of diabetes, influences cancer cell metabolism and induces shifts from glucose metabolism to fatty acid oxidation, which has been shown to decrease immune cell infiltration, polarization, and activation [12–14]. Studies have demonstrated that despite increased uptake of glucose, hyperglycemia is regulated through insulin receptor signaling and alone may not increase tumor growth [14, 15]; therefore, glucose metabolism may not be the primary driver of cancer growth and progression in the context of obesity and diabetes [16, 17]. Hyperglycemic-induced alterations in glucose metabolism create unique challenges in cancer detection and monitoring. Positron emission tomography (PET) imaging with fluorodeoxyglucose (FDG), the clinical gold-standard for diagnostic PET imaging, utilizes the fact that tumors readily metabolize glucose to distinguish tumor burden from normal metabolism in other tissues [18]. Considering the influence of hyperglycemia on blood glucose, this condition could affect the measurement of metabolic activity in tumors and potentially hinder identification of cancerous lesions.

In addition to dysregulating metabolism, obesity and T2D are known to influence the immune system and induce further inflammation [3, 19–23]. Adipose tissue, in the context of obesity, secretes pro-inflammatory cytokines like IL-6 or TNF-α and hormones like leptin, ghrelin, and adiponectin, which regulate hunger and fat deposition [24, 25]. These adipokines contribute to the chronic inflammatory state and dysregulated appetite and fat storage observed in obesity [22, 25]. Hyperglycemia alters innate immune responses including inhibition of NK cells and neutrophils and recruitment of macrophages [23, 26–28]. Further, obesity has been reported to enhance the accumulation of anti-tumoral, pro-inflammatory Th1 CD4 + and CD8 + T cells which contribute to T2D pathogenesis by inhibiting their ability to secrete effector molecules and cytokines which recruit other anti-tumor populations. This inhibition decreases immune activation, thereby suppressing effector functions which mediate tumor killing [23, 29, 30].

Importantly, PET imaging can be used to non-invasively visualize molecular markers of inflammation, immune populations, and glucose metabolism both systemically and within the tumor microenvironment [31]. Specifically, PET tracers are being investigated to visualize innate immune populations such as CD206 + macrophages and neutrophils [32, 33]. Radiopharmaceuticals targeting adaptive immune populations are also being developed including those for CD8 T cells, granzyme B + effector cells (both NK cells and CD8 + T cells), and CD4 T cells [34–36]. Molecular imaging approaches allow for comprehensive, full-body imaging which enables characterization of the tumor, important metabolic tissues, and patient-specific interactions related to immune activation and metabolism. With multiple intertwined diseases that may affect therapeutic response and progression, having a full-body approach for characterizing multiple organs and systemic effects is important. Characterizing metabolism and immune differences induced by T2D will inform on the inflammatory states that contribute to worse outcomes for cancer patients. Imaging data can then be utilized to identify biomarkers of response or resistance unique to each patient, thereby informing treatment regimens and allowing for treatment personalization. The goal of this study is to utilize advanced molecular imaging to non-invasively characterize differences in metabolism and presence of innate and adaptive immune populations in diabetic obese mice with breast cancer.

## Materials and Methods

### Cell Culture

E0771-luciferase + murine mammary carcinoma cells were purchased from ATCC (#CRL-3461) and grown in RPMI (Gibco #11,875,085) supplemented with FBS (Gibco #A5670801) to 70–80% confluence.

### Animal Model

All experiments were performed in accordance with UAB’s IACUC under APN 21655 and described in Supplemental Fig. 1. 5–6-week-old female C57BL6/J mice (Jackson Labs) were placed on a low-fat or high fat diet (Research Diets #129450ji, #12942ji). High-fat diet fed animals were treated with low-dose streptozotocin (40 mg/kg, Enzo Life Sciences #ALX-380–010-G001) via intraperitoneal injection to induce type 2 diabetes. This group, referred to as diabetic obese or abbreviated as HFD+STZ, has been previously described [10] and further expanded in the Supplemental Materials. Animals were inoculated with 1 × 10^5^ E0771-luc + cells in the right 4th mammary fat pad roughly 2 weeks prior to imaging and biological validation studies.

### BLI Imaging and Analysis

D-luciferin (GoldBio #115,144–35-9) was administered via intraperitoneal injection 10 min prior to image acquisition. BLI imaging was performed weekly on the IVIS Lumina III and analysis was performed using Living Image software (Revvity).

### Radiotracer Synthesis and Labeling

[^18^F]-FDG was purchased from a commercially available source. [^18^F]-DPA-714 [37, 38], [^68^Ga]Ga-GZP [39, 40], and [^68^Ga]Ga-RP832c [32] were produced as previously reported and described in the Supplemental Materials.

### PET/CT Imaging and Analysis

Tumor-bearing mice were imaged with [^18^F]-FDG, [^68^Ga]Ga-GZP, [^68^Ga]Ga-RP832c, and [^18^F]-DPA-714 within 5 days of each other, as shown in Supplemental Fig. 1. Radiopharmaceuticals were injected intravenously prior to imaging. Image acquisition consisted of 20-min static PET scan (SOFIE) followed by a 5-min CT for anatomical reference. Image analysis was performed (VivoQuant, Invicro) to obtain SUV values and frequency histograms for tissues of interest.

### Flow Cytometry

E0771 mammary tumors were harvested and dissociated into single cells using gentleMACS (Miltenyi Biotec #130–096–730). Cells were stained with commercially available fluorophore-conjugated antibodies for immune phenotyping, collected using BD LSRII, and analyzed in FlowJo software (TreeStar Inc). Additional details including antibody dilution and catalog numbers can be found in Supplemental Materials.

### Statistical Analysis

Independent t-tests were used to compare differences between groups for imaging data, flow cytometry, bioluminescence, body weight, and blood glucose. Pearson correlations were used to compare imaging with biological validation. Kolmogorov–Smirnov tests were used to compare frequency histograms. Sample sizes were determined using analysis with 90% power and α = 0.05. *P* values < 0.05 were considered significant.

## Results

### Induction of T2D and Obesity Correlates with Increased Tumor Volume

Mice fed HFD and treated with low-dose STZ have significantly increased blood glucose (BLG), indicative of hyperglycemia. Fasting BLG was monitored once weekly (Supplemental Fig. 2A). At the time of imaging (week 10), BLG of diabetic obese mice was significantly increased compared to control mice on the low-fat diet (LFD) (Supplemental Fig. 2B *p* = 0.0098). Body weight increased over time for both groups and is significantly increased in obese diabetic animals prior to tumor inoculation. However, body weights tend to decrease following tumor inoculation and was not significantly different for tumor-bearing diabetic obese animals compared to controls at the time of PET imaging studies (Supplemental Fig. 2C-D *p* = 0.19). Bioluminescence (BLI) imaging at week 10 shows increased viable tumor burden in diabetic obese animals compared to their control counterparts (Supplemental Fig. 2E-F *p *= 0.04).

### Glucose Metabolism is Altered Systemically in Diabetic Obese Tumor-Bearing Mice

**Fig. 1:**
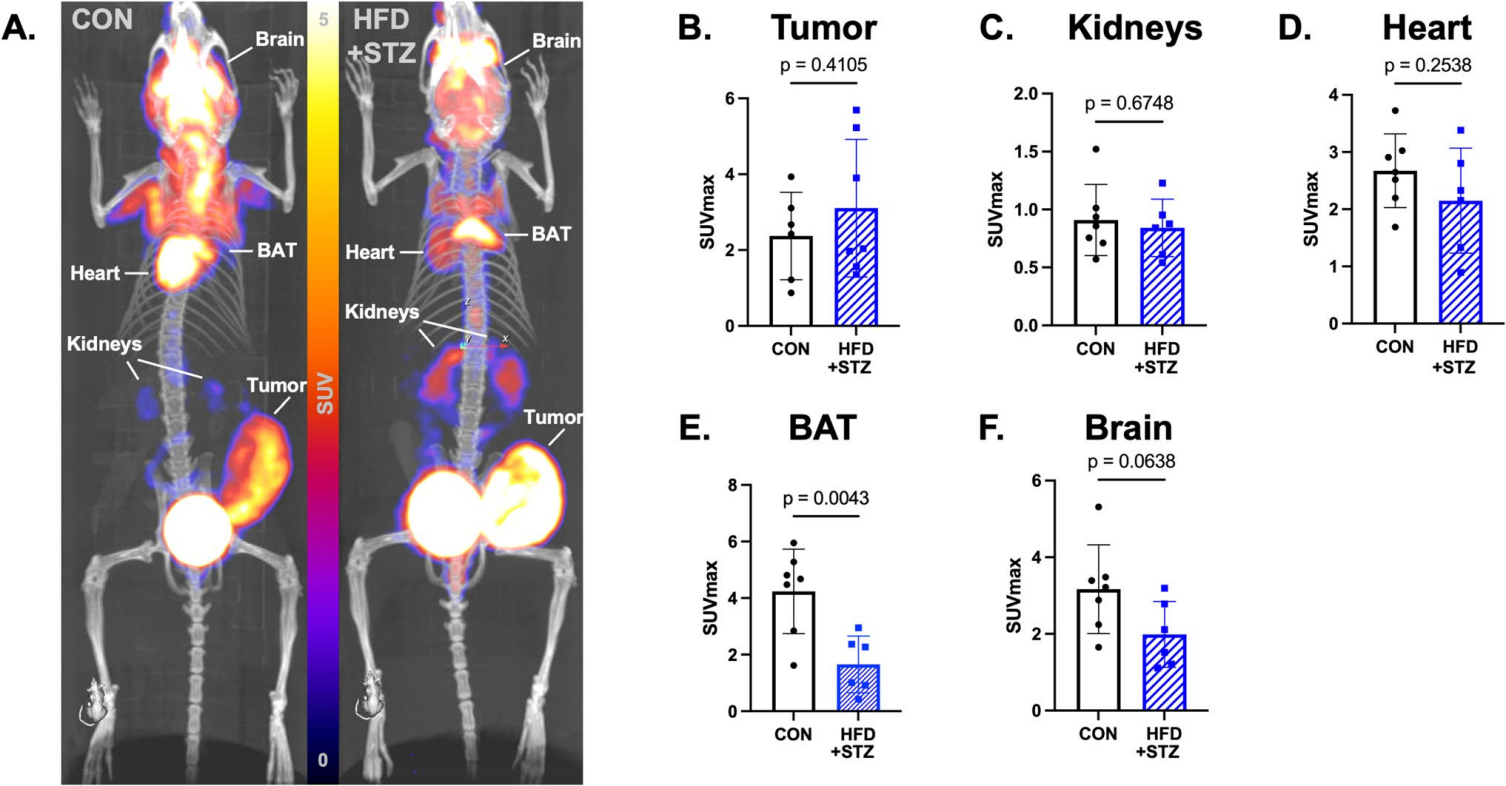
T2D and obesity alters glucose metabolism in E0771 tumor-bearing mice. **A**. Representative FDG PET maximum intensity projection (MIP) images of E0771 tumor-bearing mice from the control (left, CON) and diabetic obese (right, HFD+STZ) groups. **B**. Quantification of FDG SUV_max_ in tumors of diabetic obese mice (*p* = 0.41). **C**. Kidney FDG SUV_max_ between control and diabetic obese mice (*p* = 0.67) **D**. There is no difference in heart FDG SUV_max_ between control and diabetic obese animals (*p* = 0.25) **E**. SUV_max_ is significantly decreased in the brown adipose tissue (BAT) of diabetic obese mice (*p* = 0.0043). **F**. FDG SUV_max_ is decreased in the brain of diabetic obese mice and trends toward significance (*p* = 0.063)

Representative FDG PET maximum intensity projection images of E0771 tumor-bearing mice from the control (left) and diabetic obese (right) groups shows systemic differences in glucose metabolism (Fig. 1A). Quantification of FDG SUV_max_ shows no significant difference in tumor glucose metabolism (Fig. 1B *p* = 0.41), no significant difference in kidney FDG uptake (Fig. 1C *p* = 0.67), and no significant difference in FDG SUV_max_ in the heart between control and diabetic obese animals (Fig. 1D *p* = 0.25) SUV_max_ is significantly decreased in the brown adipose tissue (BAT) of diabetic obese mice compared to controls (Fig. 1E *p* = 0.0043). FDG SUV_max_ is trending towards decreases in the brain of diabetic obese mice compared to controls (Fig. 1F *p* = 0.063). Overall, glucose metabolism, as measured by FDG PET, is differentially influenced by obesity and T2D.

### Diabetic Obese Mice Have Decreased Glucose Metabolism and Immune Changes in the Brain

**Fig. 2.**
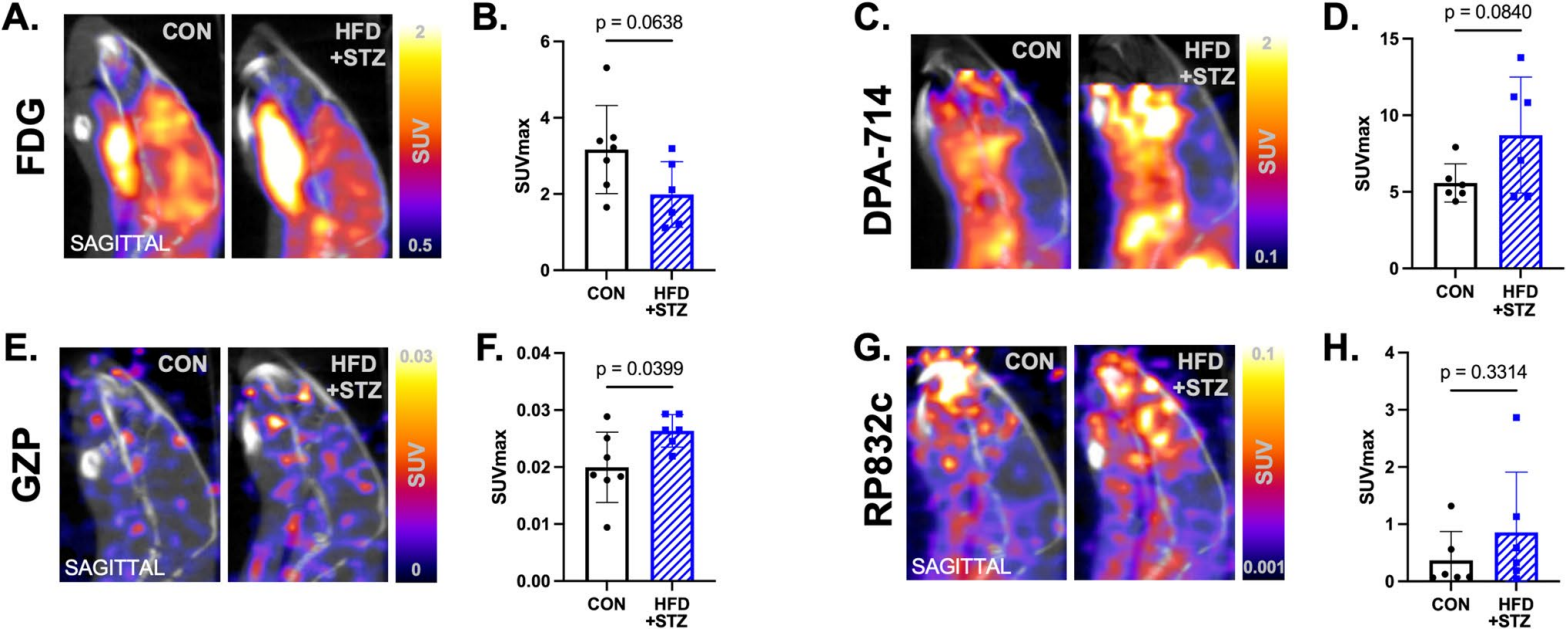
T2D and obesity alter inflammation, metabolism, and immune changes in the brain. **A**. Representative FDG PET images showing the sagittal view of the brain in control and diabetic obese mice. **B**. Diabetic obese mice have decreased FDG SUV_max_ uptake compared to control counterparts (*p* = 0.063). **C**. Representative TSPO PET images showing the brain in sagittal view. **D**. Diabetic obese mice have increased brain inflammation, as measured by TSPO SUV_max_, compared to control mice (*p* = 0.084). **E**. Representative GZP PET images showing the sagittal view of the brain. **F**. Diabetes and obesity significantly increases effector cell activation via GZP SUV_max_ (*p* = 0.039). **G**. Representative CD206 PET images showing the sagittal view of the brain. **H**. Diabetes and obesity increase CD206 SUV_max_, but not significantly (*p* = 0.33)

Hyperglycemia associated with T2D has been observed to alter cognitive function by inducing inflammation, altering metabolism, and immune changes in the brain [41–43]. Representative FDG PET images show the sagittal view of the brain in control and diabetic obese mice with quantification showing lower, trending toward significant, FDG SUV_max_ uptake compared to control counterparts (Fig. 2A-B *p* = 0.063). Representative DPA-714 PET images show the sagittal view of control and diabetic obese mice. Diabetic obese mice have trending increased brain inflammation, as measured by SUV_max_, compared to control mice (Fig. 2C-D *p* = 0.084). Diabetic obese mice have significantly higher adaptive immune activation of GZP + effector cells which is seen in representative images and in quantitative comparison of SUV_max_ (Fig. 2E-F *p* = 0.039), but have no differences in RP832c + innate immune presence compared to control. (Fig. 2G-H *p* = 0.33). Hyperglycemia alters glucose metabolism, increases neuroinflammation, and alters immune populations in brain tissue.

### Diabetic Obese Mice Have Alterations in Tumor-Immune Populations

**Fig. 3:**
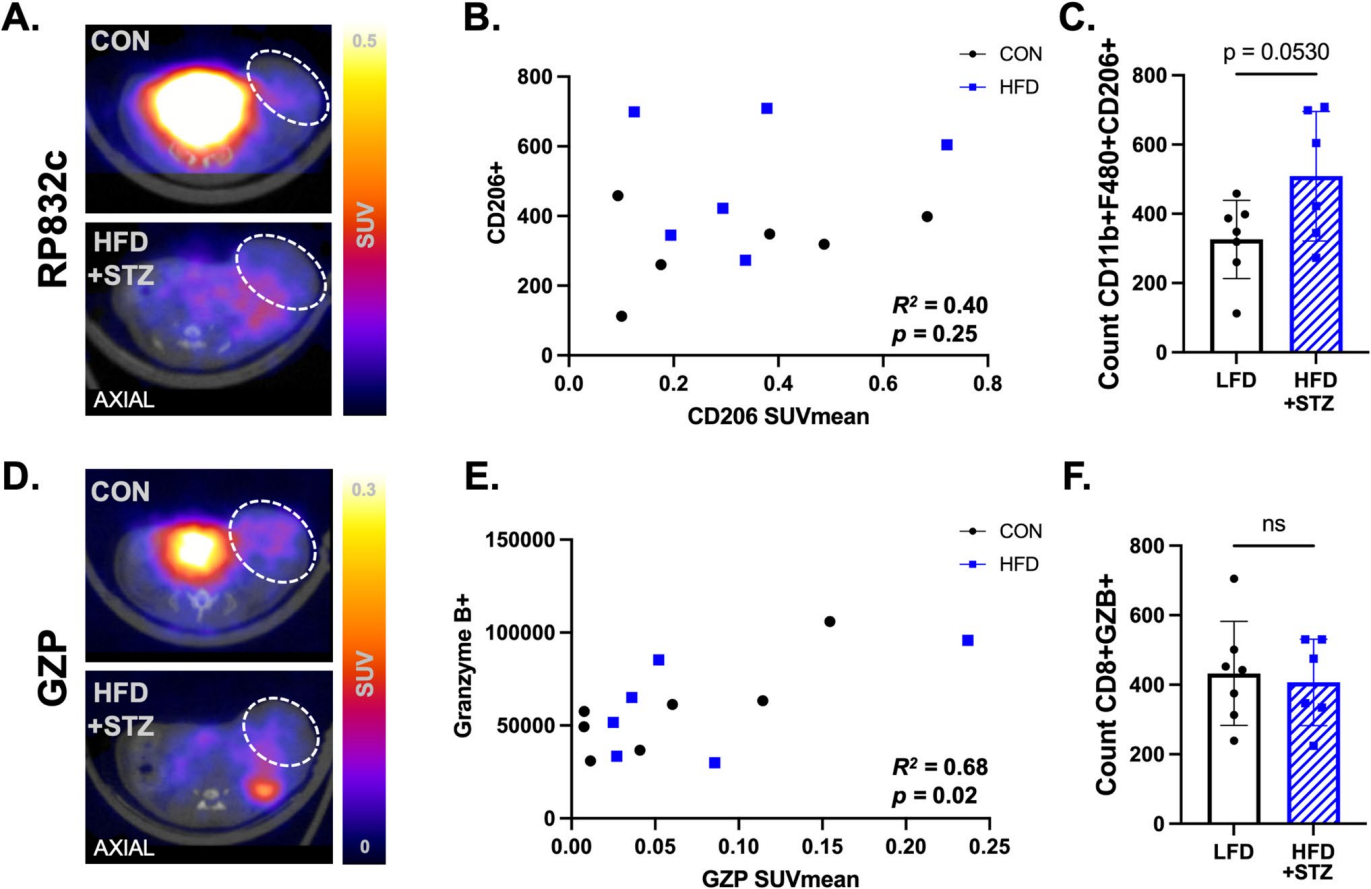
T2D and obesity induces a suppressive tumor-immune microenvironment. **A**. Representative CD206 PET images showing the axial view of tumors (white circle) in control and diabetic obese animals. **B**. CD206 SUV_mean_ and counts of CD206 + cells from flow cytometry are moderately correlated (*R*^*2*^ = 0.4, *p* = 0.25). **C**. Count of CD206 + macrophages in diabetic obese mice compared to controls (*p* = 0.053). Populations are gated on VIA > CD45 + > CD11b + F4/80 +. **D**. Representative GZP PET images showing the axial view of tumors (white circle) in control and diabetic obese animals. **E**. GZP SUV_mean_ and counts of granzyme B + cells from flow cytometry are strongly, significantly correlated (*R*^*2*^ = 0.68, *p* = 0.02). **F**. Biological validation via flow cytometry shows Granzyme B + CD8 + cell count between control and diabetic obese mice (*p* = 0.74). Populations are gated on VIA > CD8 + > GZB+

Mammary fat pad tumors of diabetic obese mice have trending increased CD206 + populations and no significant difference in GZB + effector cells compared to controls, suggestive of a potentially suppressed immune phenotype within the tumor. RP832c and GZP PET were used to non-invasively quantify innate and adaptive immune populations within mammary tumors. Representative RP832c PET images showing the axial view of tumors in control and diabetic obese animals (Fig. 3A). RP832c SUV_mean_ and counts of CD206 + cells from flow cytometry are moderately, but not significantly correlated (Fig. 3B R^*2*^ = 0.4, *p* = 0.25). Counts of CD206 + macrophages by flow cytometry trend higher in diabetic obese mice compared to controls, yet are not significantly different (Fig. 3C *p* = 0.053). Representative GZP PET images showing the axial view of tumors (white circle) in control and diabetic obese animals (Fig. 3D). GZP SUV_mean_ and counts of granzyme B + effector cells from flow cytometry are strongly, significantly correlated (Fig. 3E R^*2*^ = 0.68, *p* = 0.02), yet there are no significant differences in CD8 + granzyme B + effector cell count between control and diabetic obese mice (Fig. 3F *p* = 0.74).

### Diabetes and Obesity also Increase Innate and Adaptive Immune Presence in Highly Metabolic Tissues Including the Quadricep Muscle, Heart, and Bat

**Fig. 4:**
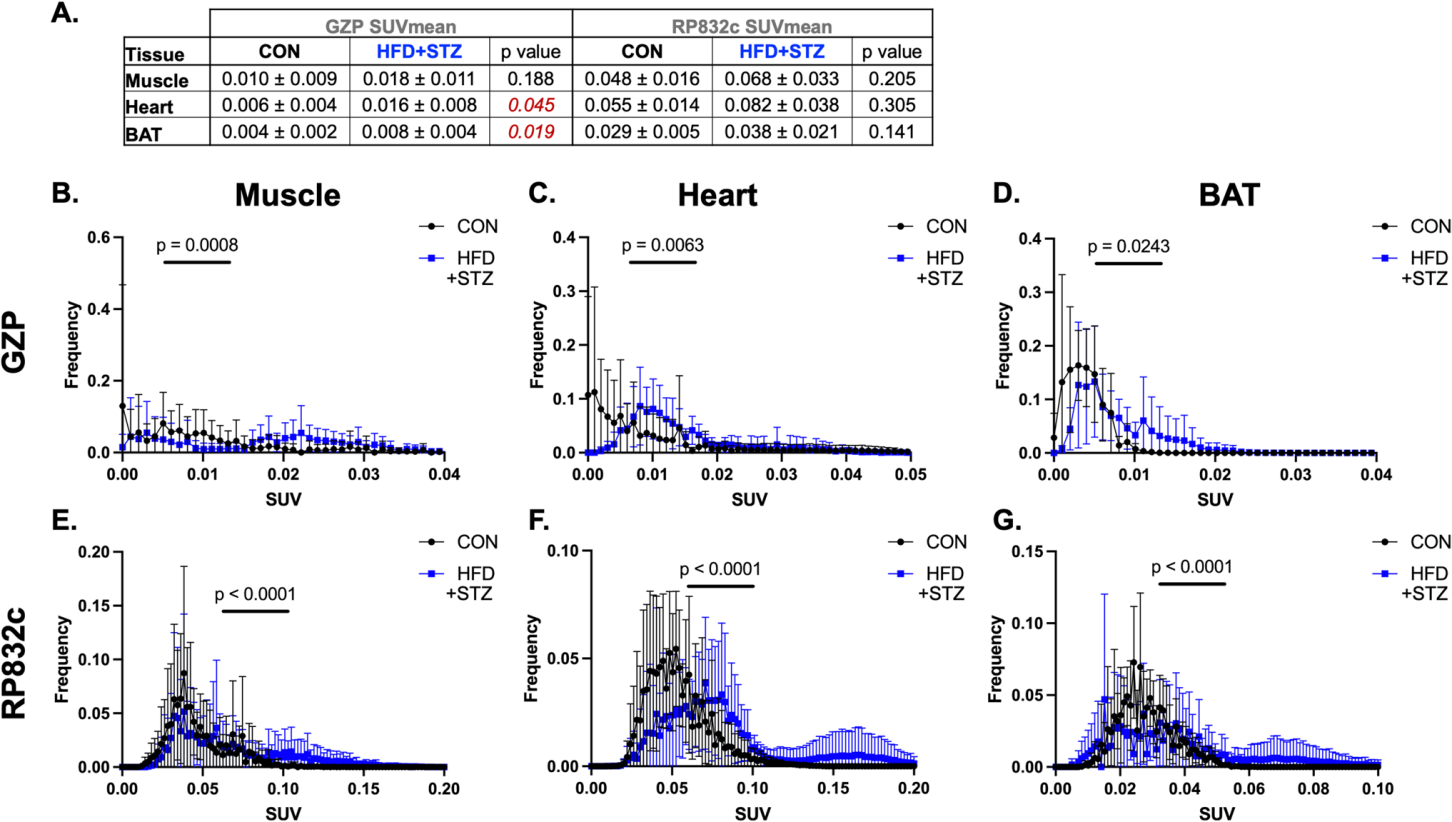
T2D and obesity alters innate and adaptive immune populations in metabolic tissues including muscle, heart, and brown adipose tissue. **A**. Table showing GZP SUVmean and RP832c SUVmean values for the muscle, brown adipose tissue (BAT), and heart tissues of control and diabetic obese mice. Granzyme B PET signal distribution is significantly different in diabetic obese mice compared to controls in muscle (**B**. *p* = 0.0008), heart (**C**. *p* = 0.006), and BAT (**D**. *p* = 0.024). RP832c PET signal distribution is significantly different in diabetic obese mice compared to controls in muscle (**E**. *p* < 0.0001), heart (**F**. *p* < 0.0001), and BAT (**G**. *p* < 0.0001)

Quantification of GZP SUV_mean_ (left) and RP832c/CD206 (right) for the muscle, heart, and BAT are shown in Fig. 4A. We observed that both uptake of GZP is increased in the heart and BAT (Fig. 4A *p* = 0.045 and *p* = 0.019) and the distributions are significantly different (Fig. 4B *p* = 0.006 and *p* = 0.024). Though GZP,SUV_mean_ is not significantly different in the muscle of diabetic obese animals compared to control, the distribution of GZP is significantly different (Fig. 4B *p* = 0.0008). Distributions show diabetic obese mice have increased effector molecule secretion, suggesting increased inflammation and potential hyperactivation of immune cells. We observed no significant differences in RP832c SUV_mean_ for muscle, heart, or BAT (Fig. 4A). However, the distribution of RP832c PET signal is significantly different in diabetic obese mice compared to controls in muscle (Fig. 4E *p* < 0.0001), heart (Fig. 4F *p* < 0.0001), and BAT (Fig. 4G *p* < 0.0001). Differences in RP832c and GZP PET signal support the suggestion that diabetic obese mice have increased inflammation and increased tissue remodeling systemically.

## Discussion

FDG PET imaging is an important cancer diagnostic tool, which can be heavily confounded by obesity and diabetes. Adiposity, chronic inflammation, and metabolic dysfunction reduce the specificity of FDG PET in distinguishing cancerous lesions from pathophysiological changes associated with these conditions. This is confirmed as numerous studies show increased base levels of FDG in diabetic obese mice compared to lean controls [44–47]. We observed significant changes in the distribution of FDG uptake within the tumors of our diabetic obese model, suggesting increased intratumoral heterogeneity. We also identified that T2D induces systemic alterations in glucose metabolism in important metabolic and regulatory tissues including the brain, heart, and brown adipose tissue. Although we did not perform cognitive analyses, there is a known relationship between PET signal in systemic tissues and cognition. Studies have shown that FDG uptake was decreased in patients with hyperglycemia and increased in muscle and adipose tissue [45]. This condition has also been reported to alter mood state and cognitive function and decrease cardiac output [41–43]. Insufficient glucose processing and metabolism in these key tissues may contribute to the pathologies observed in clinical cases of hyperglycemia. Further, systemic alterations in glucose metabolism can impact tumor growth and response to therapies.

DPA-714, which binds the translocator protein (TSPO) on the outer mitochondrial membrane, is highly expressed on activated microglia. Increased TSPO expression is commonly used as a marker of neuroinflammation in traumatic brain injury and neurodegenerative disorders including Alzheimer’s disease and Parkinson’s disease [38], but is currently being investigated as a measure of systemic inflammation for other applications including cancer [48–50]. Studies have shown that DPA-714 has different pharmacokinetics across various animal models and thus can have differential uptake in peripheral organs [50]. We observed significant differences in DPA-714 uptake in the brain of hyperglycemic animals compared to controls, but observed no other differences in systemic tissues. However, given the temporal nature of inflammation, we believe systemic changes in DPA-714 may be observed at later timepoints following induction of our model and not best captured by a single imaging timepoint. The effects of T2D and chronic inflammation in systemic tissues may take longer to emerge and be detected through molecular imaging.

Metabolic disorders, including obesity and T2D, are chronic inflammatory conditions characterized by atypical cytokine production, increased circulation of pro-inflammatory proteins and hormones, and recruitment of pro-tumoral immune populations which heavily influence cancer treatment efficacy [19, 20]. Chronic inflammation can directly increase cell proliferation and promote resistance to cell death or have indirect influence on tumor growth by stimulating and recruiting pro-tumor stromal and lymphocytes, like fibroblasts and macrophages, to the tumor microenvironment [51]. Tumor-infiltrating lymphocytes and cancer cells release many inflammatory mediators including VEGF, IL-6, and GM-CSF, which further activities and recruitments immunosuppressive immune cells. Constant and excess secretion of inflammatory mediators leads to accumulation of immune-suppressive phenotypes and secreted factors which sustain tumor growth and alter systemic metabolism [52, 53]. We observed that diabetic obese tumors had trending increases in CD206 + macrophages, which are known to have pro-tumoral functions. This finding corroborates most literature, which suggests that hyperglycemia increases CD206 + populations in the tumor microenvironment [10, 54, 55]. Importantly, immune-specific imaging can provide an understanding of how chronic inflammatory conditions change the tumor-immune microenvironment and ultimately influence response to therapy.

We also observed systemic alterations in the innate and adaptive immune populations in multiple highly metabolic tissues including the muscle, heart, and brown adipose tissue, which may contribute to chronic inflammation. These tissues have many mitochondria and are key regulators of skeletal muscle function, inflammatory responses, and thermogenesis [56]. Since mitochondria are known to utilize glucose metabolism, we believe there may be a more complex dynamic in glucose biodistribution between normal physiological processes and immune cells in these tissues. Of note, studies have observed that increased granzyme B in circulation was associated with T2D diagnosis and contributes to pancreatic islet destruction, both of which are correlated with systemic inflammation [57, 58]. Increased CD206 + and GZP + effector cell presence in these tissues may be attributable to chronic inflammation and potentially dysfunctional autoreactive immune populations. PET imaging reveals diabetes-induced systemic differences in innate and adaptive immune populations. Overall, our studies show that T2D induces shifts in the tumor-immune microenvironment, alters systemic glucose metabolism, and induces inflammation in important metabolic tissues.

One opportunity for expansion of this study is to include additional tumor models with a range of anticipated immune distribution. The E0771 tumor model is considered immunogenically “hot”, and the differences we observed in immune activation and infiltration may be attributed to the innate Th1-skewed responses generated by C57BL/6 mice compared with other mouse strains [59]. There are also natural limitations within peptide-based imaging agents including GZP and RP832c, including low binding affinity, stability, and bioavailability [32, 35]. For these targets specifically, various cell types express and secrete these molecules. Granzyme B is a serine protease typically secreted by CD8 T cells, CD4 T cells, and NK cells; however, we quantified this expression non-invasively with PET imaging and therefore the functionality of each cell type was not evaluated. Similarly, CD206 is expressed in low levels on dendritic cells, fibroblasts, and Kupffer cells within the liver. However, CD206 expression is much higher in tumor-associated macrophages compared to other populations, therefore increased specificity for imaging macrophages. PET imaging can provide a non-invasive picture of the immune and metabolic landscape both within the tumor microenvironment and systemically, which can be used to characterize multiple organs in cancer and chronic inflammatory diseases.

## Conclusion

Overall, PET imaging reveals differences in metabolism and immune cell activation at the molecular level both within the diabetic obese TNBC tumor microenvironment and systemically. Via RP832c and GZP PET, we observed significant changes in immune landscapes of metabolic and regulatory tissues including the brain, heart, muscle, and brown adipose tissues. Imaging of diabetic obese tumors shows suppressed immune activation, as indicated by increased CD206 expression. Translationally, understanding systemic differences in metabolism and immune cell activation and infiltration can provide information on how tumors may respond to therapies, specifically in chronic inflammatory conditions like obesity and diabetes.

## Supplementary Information

Below is the link to the electronic supplementary material.

Supplementary file1 (DOCX 521 KB)

## Data Availability

Data is available upon request to the corresponding author.
