# Supplementary material for: PET Imaging of Diabetes-Induced Alterations in Metabolism and Immune Activation
Source: Mol Imaging Biol. 2025 Aug 12;27(5):839–48. doi: 10.1007/s11307-025-02027-y (PMC12628390; doi:10.1007/s11307-025-02027-y)
Supplement: Supplementary file 1 — Supplementary file1 (DOCX 521 KB) [file 11307_2025_2027_MOESM1_ESM.docx]

**SUPPLEMENTAL MATERIALS AND METHODS**

**Cell culture**

E0771-luciferase+ murine mammary carcinoma cells were purchased from American Type Culture Collection (ATCC, Manassas, VA, #CRL-3461). E0771 cells were cultured in Roswell Park Memorial Institute (RPMI) 1640 Medium (D-Glucose 2 gm/L, Life Technologies, Carlsbad, CA, #11875085) supplemented with 10% heat-inactivated fetal bovine serum (FBS; Gibco, Life Technologies, Carlsbad, CA #A5670801) under puromycin (Millipore Sigma, Burlington, MA #MABE343) selection. Cells were grown at 37ºC with 5% CO_2_ and cultured to 70-80% confluence.

**Animal model**

All experiments involving animal procedures including tumor inoculation, imaging, euthanasia, and biological validation were performed in accordance with UAB’s Institutional Animal Care and Use Committee (IACUC) under Animal Protocol Number (APN) 21655. 5-6 week-old female C57BL6/J mice (Jackson Labs, Bar Harbor, ME) were placed on either a low-fat diet composed of 10% kcal from fat (**Supplemental Fig. 1**, n=7, Research Diets, New Brunswick, NJ, #129450ji) or a high-fat diet (HFD) composed of 60% kcal from fat (n=6, Research Diets, #12942ji). Diets were changed twice weekly. Animals were treated with low-dose streptozotocin (40 mg/kg, Enzo Life Sciences, Farmingdale, NY, #ALX-380-010-G001) via intraperitoneal (IP) injection at days 15-17 and 43-45 following diet initiation to induce type 2 diabetes, as previously described [11]. Animals receiving high-fat diet and streptozotocin are referred to as HFD or diabetic obese animals within figures and the manuscript. Fasting blood glucose was monitored at baseline and weekly starting at day 21 of diet initiation. Body weights were measured once weekly and every imaging day. After 48 days on the diet, animals were inoculated with 1x10^5^ E0771-luciferase+ cells in the right 4^th^ mammary fat pad. Tumor volume was monitored twice weekly via calipers and once weekly with bioluminescence to determine tumor cell viability beginning at day 7 post-inoculation. During imaging studies and tumor inoculation, animals were anesthetized via 2% isoflurane inhalation. For biological validation studies, animals were euthanized via over-administration of isoflurane followed by cervical dislocation, and tumors were extracted for flow cytometry.

**BLI imaging and analysis**

Bioluminescence imaging (BLI) (IVIS Lumina III, Perkin Elmer, Waltham, MA) was performed once weekly at 7 days post-tumor inoculation to quantify changes in tumor cell viability and growth. 100 µL of D-luciferin (GoldBio, Olivette, MO, #115144-35-9) was administered via IP injection and 5-minute image acquisition was performed 10 minutes post-injection. Analysis and quantification were performed using Living Image^TM^ software (Revvity, Waltham, MA). Regions of interest (ROI) were drawn on tumors and background to assess tumor viability.

**Radiotracer synthesis and labeling**

**[^18^F]-FDG** [^18^F]-fluorodeoxyglucose (FDG) was purchased from PETNET Solutions Inc (Siemens), a commercially available source which produces clinical and preclinical grade radiotherapeutics.

**[^18^F]-DPA-714/TSPO** F-18 was produced from a TR24 cyclotron facility at the University of Alabama at Birmingham and radiolabeled with DPA-714 or N, N-diethyl-2-[4-phenyl]-5,7-dimethylpyrazolo[1,5-a]pyrimidine-3-acetamide, a selective ligand to image translocator protein (TSPO) expression, as previously reported [48, 49].

**[^68^Ga]Ga-GZP** Ga-68 was obtained from a commercially available ^68^Ge/^68^Ga generator (Eckert & Ziegler, MA, USA) by elution via 0.1 M HCl at a rate of 1 mL/minute. Eluent was brought to pH 4.5 using 1 M sodium acetate, and combined with 50 µg of NOTA-GZP (NOTA–βAla–Gly–Gly–Ile–Glu–Phe–Asp–CHO, granzyme B peptide). The reaction vial was incubated for 10 minutes at 37ºC while shaking at 300 rpm on a thermomixer. Elution of the final product from a C18 Sep-Pak mini cartridge (Waters #WAT023501) was performed using 200 µL of 70% EtOH followed by 200 µL of dPBS as previously reported [50, 51]. Radiolabeling efficiency was determined using instant thin layer chromatography (iTLC) on an AR-2000 Imaging Scanner (Eckert and Ziegler) and radiochemical yields of >95% purity were used for *in vivo* studies.

**[^68^Ga]Ga-RP832c/CD206** Ga-68 was obtained from a commercially available ^68^Ge/^68^Ga generator (Eckert & Ziegler) by elution via 0.1 M HCl at a rate of 1 mL/minute through a strong cation exchange cartridge (Agilent Bond Elut SCX Cartridges, 100 mg, 40 µM #12102013). 200 µL of 5 M NaCl/HCl was used to isolate ^68^GaCl_3_ from the cartridge. 500 µCi of Ga-68, 1 M sodium acetate (pH 4.5), and 10 µg of DOTA-RP832c (DOTA–Arg–Trp–Phe–Gly–Gly–Phe–Lys–Trp–Arg–CHO, macrophage mannose receptor) were combined and the reaction incubated for 15 minutes at 95ºC while shaking at 300 rpm on a thermomixer as previously reported [41]. Radiolabeling efficiency was determined using iTLC on an AR-2000 scanner and radiochemical yields of >95% purity were used for *in vivo* studies.

**PET/CT imaging and analysis**

Tumor-bearing mice were imaged with [^18^F]-FDG, [^68^Ga]Ga-GZP, [^68^Ga]Ga-RP832c, and [^18^F]-DPA-714 within 3 days of each other at 3 weeks post-tumor inoculation. To image glucose metabolism, mice were fasted overnight and given 100±20 µCi in 100 µL of [^18^F]-FDG via intravenous (IV) injection and imaging was performed 60-minutes post-injection. To image CD206+ populations, mice were given 100±20 µCi in 100 µL of [^68^Ga]Ga-RP832c via IV injection and imaging was performed 30-minutes post-injection. To image granzyme B+ effector cell population, mice were given 200±20 µCi in 100 µL of [^68^Ga]Ga-GZP via IV injection and imaging was performed 60-minutes post-injection. To image TSPO (a surrogate for neuroinflammation), mice were given 100±20 µCi in 100 µL of [^18^F]-DPA-714 via intravenous injection and imaging was performed 60-minutes post-injection. Image acquisition parameters for all radiotracers includes a 20-minute static PET scan followed by 5-minute CT at 80 kVp. PET/CT image acquisition was performed using preclinical PET/CT (GNEXT, SOFIE, Culver City, CA).

Image analysis was performed in VivoQuant software (Invicro, Needham, MA). SUV was calculated using the formula SUV = C / (dose/weight) where C is the tissue radioactivity concentration, dose is the injected dose in µCi, and weight is body weight of the animal in grams. Regions of interest (ROIs) were indicated in tissue using anatomical reference and SUV uptake including mean, max, and peak was quantified. ROIs were also drawn in the tumor, brain, kidneys, brown adipose tissue (BAT) atrium (heart), and contralateral quadricep (muscle) of each animal. Histogram data from tumors was analyzed to quantify the uptake in the highest expressing regions (top 10% of voxels), similarly to other reported methods [52, 53].

**Flow cytometry**

Mammary tumors from E0771 tumor-bearing mice were harvested, and immediately placed in C-Tubes (Miltenyi Biotec, Auburn, CA, #130-093-237) containing DMEM (Life Technologies) with tumor dissociation enzymes from the mouse tumor dissociation kit (Miltenyi Biotec, #130-096-730). Tumors were dissociated using the Miltenyi gentleMACS Octo Dissociator with heaters following manufacturers protocol and using the program 37C_m_TDK_2. Following tissue dissociation, cells were washed with 1X phosphate-buffered saline (PBS) with 2% FBS, centrifuged, and red blood cells were lysed in ACK lysis buffer for 30 seconds, followed by two washes in 1X PBS with 2% FBS. Cells were then counted and stained with the viability dye eFluor450 (ThermoFisher, #65-0863-14) in 1X PBS for 20 minutes on ice in the dark. Next, cells were incubated in primary conjugated antibody cocktail (BioLegend, #199901) for extracellular markers and stained for 30 minutes on ice in the dark. After extracellular staining, cells were fixed for 45 minutes at room temperature in the dark using the intracellular fixation buffer (BioLegend, #420801) and then permeabilized using the true nuclear transcription factor buffer set according to the manufacturer’s protocol. Cells were stained with a primary antibody cocktail (BioLegend, #199901) in permeabilization buffer for intracellular or nuclear markers for 30 minutes on ice in the dark. Antibodies were purchased from commercially available sources including CD11b-FITC (BioLegend, #101206), CD206-PE-Cy7 (BioLegend, #141720), F4/80-BV605 (BioLegend, #123133), Live/Dead-eFluor450 (ThermoFisher, #65-0863), CD8a-PE-Cy7 (BioLegend, #100722), Granzyme B-PE (BioLegend, #372208). The cells were washed twice between each step. Cells were resuspended in FACS buffer comprised of 5% BSA (ThermoFisher, #15561020) and 1% sodium azide (ThermoScientific, #190380050) diluted in 1X PBS and analyzed using BD LSRII analyzer. Subsequent analysis was performed using FlowJo 10.6.2 software (TreeStar Inc, Ashland, OR) and data are represented counts per one million events.

**Statistical analysis**

An independent t-test was used to compare differences between control and diabetic obese animals. Pearson correlations were used to evaluate relationships between imaging and biological validation data. Kolmogorov-Smirnov (KS) tests were performed to determine differences in frequency distributions for histogram data obtained from PET imaging. Sample sizes were determined using analysis with 90% power and α=0.05. P values < 0.05 were considered significant.


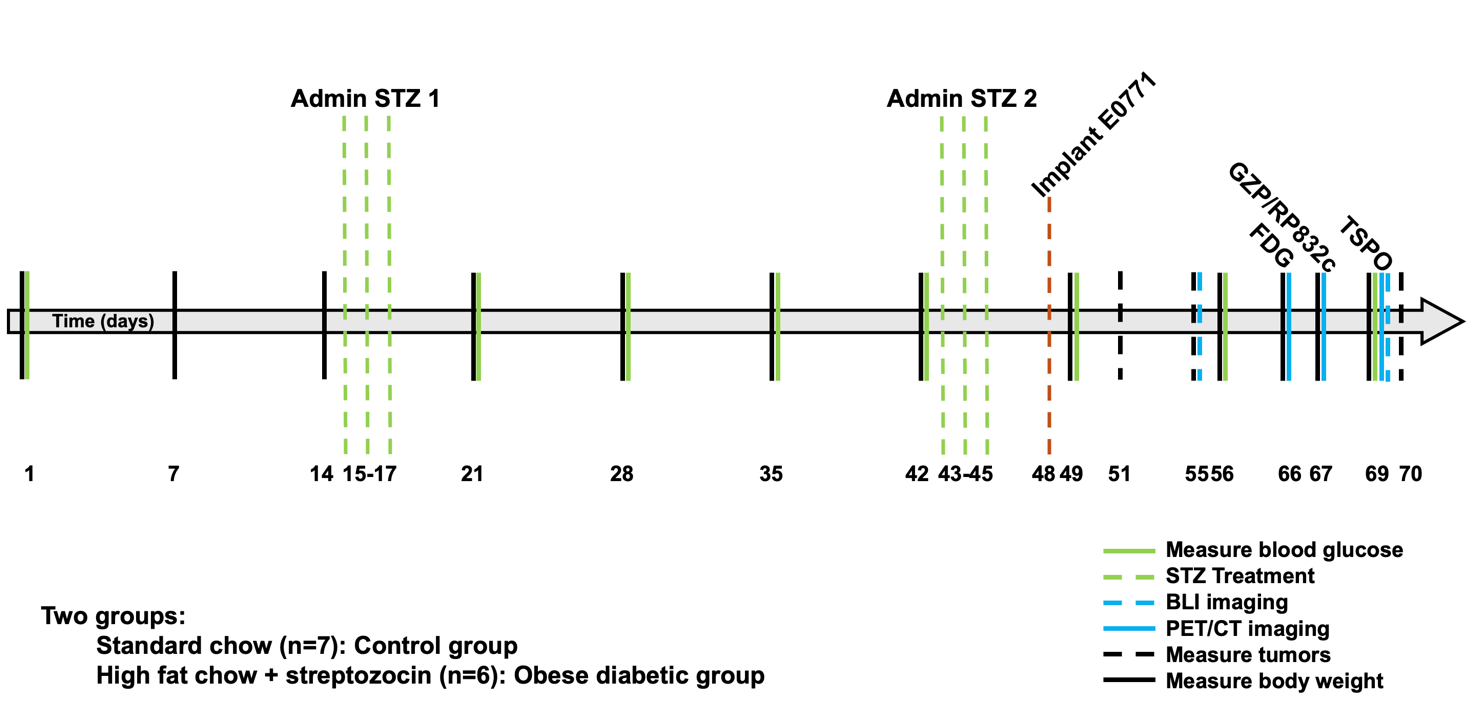


**Supplemental Figure 1. Experimental timeline of a major procedures including model development, tumor inoculation, and PET imaging.** Timeline demonstrating the generation of diabetic obese animals via administration of a high-fat diet (HFD) and low-dose streptozotocin (STZ) at 40 mg/kg. E0771-luc+ cells were inoculated into the 4^th^ mammary fat pad of female mice at 48 days post-diet initiation and monitored once weekly via bioluminescence (BLI) imaging. Beginning on day 66 following diet initiation and 18 days following tumor inoculation, upon tumors reach at least 100 mm^3^, animals underwent PET imaging. FDG PET on day 66, GZP and RP832c PET on day 67 with 6 hours in between injections for each tracer, and finally TSPO PET on day 69. The following day, animals were euthanized, and tumors were harvested for biological studies via flow cytometry.


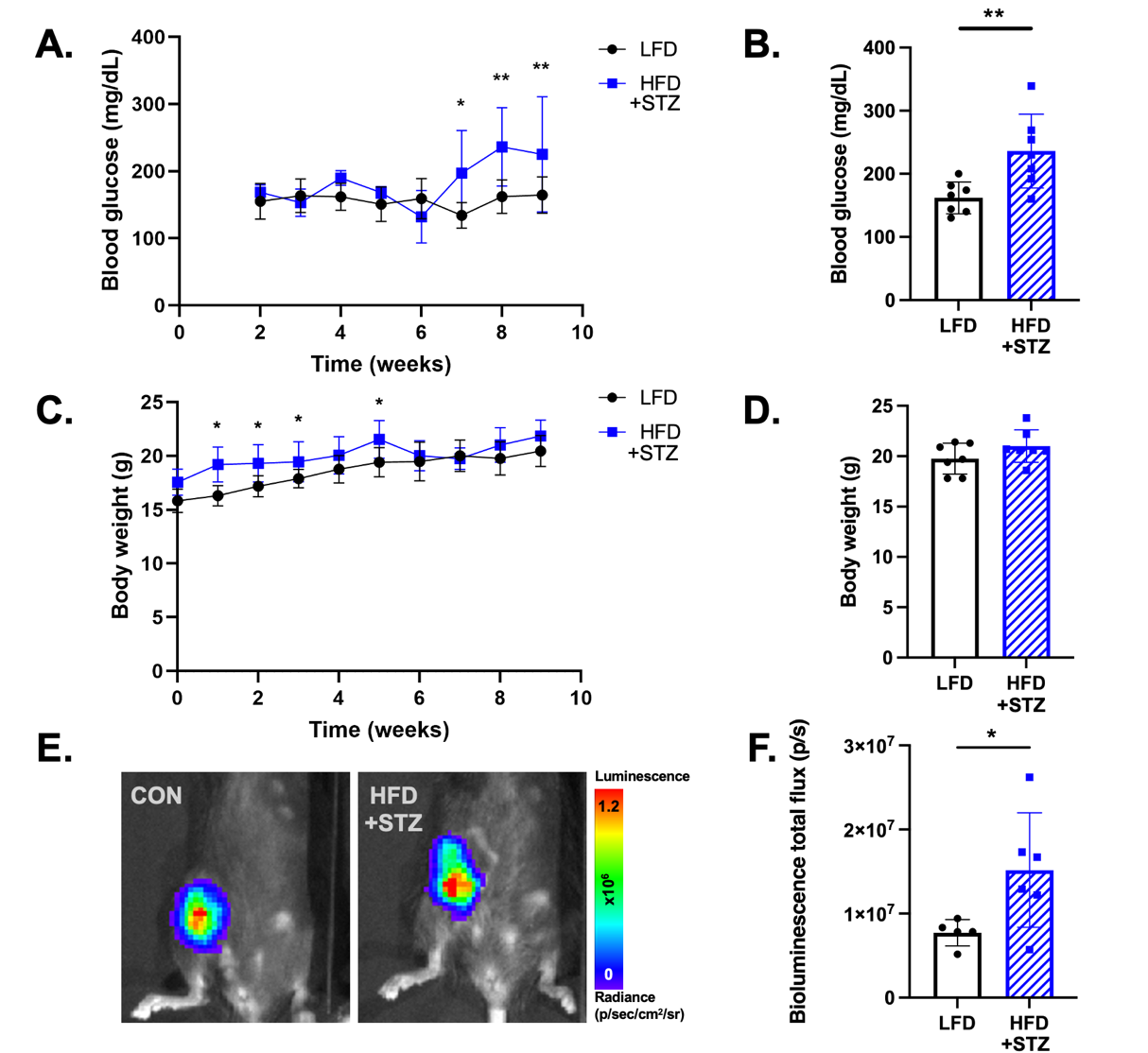


**Supplemental Figure 2. Low-dose STZ and HFD mice have significantly increased fasting blood glucose, body weight, and tumor volume compared to control.** **A.** Fasting blood glucose (BLG) increases over time for hyperglycemic mice (*p =* 0.024). **B.** BLG of hyperglycemic mice is significantly increased compared to controls during week 10 when imaging occurs (*p =* 0.009). **C.** Body weight increases over time for diabetic obese mice. **D.** Body weight is not significantly different between groups at week 10 when imaging occurs (*p =* 0.19). **E.** Representative bioluminescence (BLI) images with quantification of E0771-luc+ tumor volume from control and hyperglycemic mice. **F.** Total flux (photons per second, p/s) representing tumor volume is significantly increased for diabetic obese mice compared to control animals (*p =* 0.04).
